# Bicarbonate Resensitization of Methicillin-Resistant *Staphylococcus aureus* to β-Lactam Antibiotics

**DOI:** 10.1128/AAC.00496-19

**Published:** 2019-06-24

**Authors:** Selvi C. Ersoy, Wessam Abdelhady, Liang Li, Henry F. Chambers, Yan Q. Xiong, Arnold S. Bayer

**Affiliations:** a Los Angeles Biomedical Research Institute, Torrance, California, USA; b Geffen School of Medicine, University of California Los Angeles, Los Angeles, California, USA; c Division of Infectious Diseases, Zuckerberg San Francisco General Department of Medicine, San Francisco School of Medicine, University of California, San Francisco, California, USA

**Keywords:** antimicrobial susceptibility testing, MRSA, β-lactams, infective endocarditis, penicillin-binding proteins, sodium bicarbonate

## Abstract

Endovascular infections caused by methicillin-resistant Staphylococcus aureus (MRSA) are a major health care concern, especially infective endocarditis (IE). Standard antimicrobial susceptibility testing (AST) defines most MRSA strains as “resistant” to β-lactams, often leading to the use of costly and/or toxic treatment regimens. In this investigation, five prototype MRSA strains, representing the range of genotypes in current clinical circulation, were studied. We identified two distinct MRSA phenotypes upon AST using standard media, with or without sodium bicarbonate (NaHCO_3_) supplementation: one highly susceptible to the antistaphylococcal β-lactams oxacillin and cefazolin (NaHCO_3_ responsive) and one resistant to such agents (NaHCO_3_ nonresponsive). These phenotypes accurately predicted clearance profiles of MRSA from target tissues in experimental MRSA IE treated with each β-lactam. Mechanistically, NaHCO_3_ reduced the expression of two key genes involved in the MRSA phenotype, *mecA* and *sarA*, leading to decreased production of penicillin-binding protein 2a (that mediates methicillin resistance), in NaHCO_3_-responsive (but not in NaHCO_3_-nonresponsive) strains. Moreover, both cefazolin and oxacillin synergistically killed NaHCO_3_-responsive strains in the presence of the host defense antimicrobial peptide (LL-37) in NaHCO_3_-supplemented media. These findings suggest that AST of MRSA strains in NaHCO_3_-containing media may potentially identify infections caused by NaHCO_3_-responsive strains that are appropriate for β-lactam therapy.

## Abstract (precis)

Endovascular infections caused by methicillin-resistant Staphylococcus aureus (MRSA) are a major health care concern, especially infective endocarditis (IE). Standard antimicrobial susceptibility testing (AST) defines most MRSA strains as “resistant” to β-lactams, often leading to the use of costly and/or toxic treatment regimens. In this investigation, five prototype MRSA strains, representing the range of genotypes in current clinical circulation, were studied.

## INTRODUCTION

Staphylococcus aureus is a major bloodstream pathogen in both community-acquired and nosocomially acquired scenarios and is the leading cause of infective endocarditis (IE) in the industrialized world (1). Compounding the danger of S. aureus bloodstream infections (BSIs) is the steady rise of methicillin-resistant Staphylococcus aureus (MRSA) strains in many geographic regions in the United States (2). MRSA is a serious infectious threat, causing more than 15,000 deaths in the United States each year (3).

MRSA have high MICs that are above Clinical and Laboratory Standards Institute (CLSI) resistance breakpoints for most conventional β-lactam antibiotics, such as oxacillin, on standard antimicrobial susceptibility testing (AST) media. This finding implies a lack of efficacy of these agents in treating MRSA infections, as confirmed in selected experimental IE studies (4–6). Treatment options for MRSA are generally limited to costlier and/or more toxic drugs, such as vancomycin, daptomycin, and lipoglycopeptides, and fifth-generation cephalosporins, such as ceftaroline (7–9). In addition, great expense and effort have gone into development of such newer anti-MRSA drugs (10, 11).

AST protocols for MRSA have been standardized by the CLSI and involve growth of bacterial samples in 2% NaCl cation-supplemented, nutrient-rich Mueller-Hinton broth (CA-MHB) (12, 13). However, MHB does not accurately represent the host milieu, and MICs observed in this medium are unlikely to mirror those exhibited by MRSA within specific host microenvironments. Although research efforts have been made to effectively model the host microenvironment *in vitro* or *ex vivo* (e.g., using simulated endocardial vegetations [SEVs]) (14, 15), these models are not suitable for large-scale AST in clinical laboratories.

Recently, several groups have attempted to improve standardized AST by altering growth conditions to better reflect the host environment. For example, AST of intracellular bacteria, such as *Salmonella*, in media that models the host phagolysosome can better predict treatment outcomes in murine bacteremia models (16, 17). Growth of bacteria in Dulbecco modified Eagle medium (DMEM) (but not in standard AST media) can stimulate expression of virulence factors typically exhibited *in vivo* (18, 19). These observations led to the discovery that AST of extracellular bacteria performed in such cell culture medium (including DMEM and RPMI 1640) was a better predictor of *in vivo* treatment outcomes than standard AST media for Acinetobacter and some staphylococci (17, 20). Of note, Dorschner et al. identified the role of physiological concentrations of NaHCO_3_ in facilitating the *in vitro* killing of Escherichia coli and MRSA by host antimicrobial peptides (AMPs) (21). The carbonate molecule was capable of altering expression of a number of key regulatory genes in E. coli, including the homolog of the MRSA stress response regulator, sigma factor B (*sigB*). Finally, our lab has recently confirmed the key role of NaHCO_3_ supplementation in rendering a range of bacterial pathogens, including MRSA, as more susceptible *in vitro* to multiple antibiotics, including β-lactams (17). The predictive role of this “NaHCO_3_-responsive” phenotype, *vis-à-vis* salutary outcomes to β-lactam therapy, was corroborated in a murine bacteremia model (17).

In the present study, we focused on the activity of two β-lactam agents routinely used for treating methicillin-susceptible S. aureus (MSSA) infections against five well-characterized prototype MRSA strains, in the presence or absence of NaHCO_3_-supplemented standard media. Using this modified AST schema, we demonstrate that NaHCO_3_ supplementation increased the *in vitro* susceptibility of selected MRSA strains to both cefazolin and oxacillin (i.e., NaHCO_3_-responsive MRSA). Although these drugs are typically not recommended for the treatment of MRSA (22), we observed that they can be highly effective in treating such NaHCO_3_-responsive MRSA strains in a rabbit model of IE. In contrast, MRSA strains that were NaHCO_3_ nonresponsive *in vitro* were recalcitrant to such β-lactam therapy *in vivo*.

If verified in larger MRSA screening studies, these novel findings may potentially prompt modifications of AST for MRSA; this, in turn, could potentially guide new treatment algorithms for selected MRSA infections with β-lactam agents such as cefazolin and antistaphylococcal penicillins. Finally, mechanistically, NaHCO_3_ responsiveness correlated with the capacity of this molecule to suppress at least two key regulatory pathways intimately involved in the MRSA phenotype: *mecA*-PBP2a and *sarA* (5, 23, 24).

## RESULTS

### Effect of NaHCO_3_ on β-lactam susceptibility *in vitro*.

MIC values obtained in standard CA-MHB for the five study strains were generally within 2-fold of those obtained in CA-MHB plus 100 mM Tris (CA-MHB-Tris; see Table S1 in the supplemental material). MRSA strains 11/11 (USA300 genotype) and MW2 (USA400 genotype) displayed a substantial decrease in MICs to cefazolin and oxacillin when grown in media containing 44 mM NaHCO_3_ (Table 1). Strain MRSA 11/11 also displayed a decrease in MICs to cefazolin and oxacillin, albeit less pronounced, when exposed to a more physiologically relevant concentration of NaHCO_3_ (25 mM). In contrast, β-lactam MICs for three other prototype MRSA strains, COL (USA100 genotype), BMC1001 (USA500 genotype), and 300-111 (CC8, *spa* type 4, Iberian clone) were unaffected by exposure to NaHCO_3_ at either concentration (Table 1). Based on these two distinct phenotypes, we termed the two strains above, whose β-lactam MICs were substantially reduced by NaHCO_3_, “responsive,” while we termed the other three strains, whose β-lactam MICs were unaffected by NaHCO_3_, “nonresponsive.”

**TABLE 1:** MICs of β-lactam antibiotics against methicillin-resistant S. aureus grown in media with and without NaHCO_3_^*a*^

Treatment	Cefazolin MIC (μg/ml)					Oxacillin MIC (μg/ml)				
	MRSA 11/11	MW2	COL	BMC1001	300-111	MRSA 11/11	MW2	COL	BMC1001	300-111
CA-MHB-Tris	16	8	256	256	64	32	64	512	256	16
CA-MHB-Tris + 25 mM NaHCO_3_	4	8	512	256	32	4	32	512	256	16
CA-MHB-Tris + 44 mM NaHCO_3_	0.5	1	256	256	32	0.5	2	512	256	32
RPMI 1640 + 10% LB medium	1	2	16	2	2	1	1	64	16	4

a MIC values obtained in CA-MHB-Tris for strains MRSA 11/11, MW2, COL, and BMC1001 were within 2-fold of those obtained in standard CA-MHB (see Table S1 in the supplemental material). The MIC values for 300-111 in CA-MHB were 16 and 8 μg/ml for cefazolin and oxacillin, respectively.

In addition, several recent investigations have promoted the use of the tissue culture medium, RPMI 1640, as a more physiologic, host-mimicking media and a better predictor of *in vivo* susceptibility to various antimicrobials than standard growth media, especially for Gram-negative bacteria (20, 25). As opposed to our NaHCO_3_ assays, we found that MICs to both β-lactams were decreased in RPMI 1640 for all five of our study strains, although the effect was somewhat less substantive for strain COL (Table 1).

To evaluate whether NaHCO_3_ sensitization of MRSA to β-lactams was merely a “weak acid” effect, we investigated the impact of salicylic acid exposure on β-lactam MICs among four of our study strains. Exposure of these MRSA strains to 25 and 50 μg/ml salicylic acid, concentrations that are physiologically achievable during aspirin therapy (26), had no effect on the β-lactam MICs in any strain tested (Table S1). These data are in accord with those of Farha et al. using two other weak acid molecules, acetate and borate (27). Taken together, this finding indicates that β-lactam resensitization of some MRSA in the presence of NaHCO_3_ is not the result of a generalized weak acid effect.

**FIG 1:**
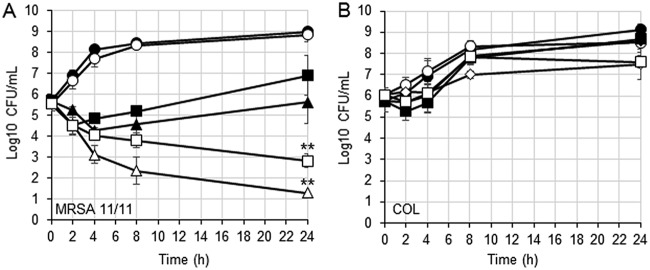
Time-kill analysis of log-phase cells grown in media with or without NaHCO_3_ . (A) MRSA 11/11; (B) COL. Growth in CA-MHB-Tris (closed symbols) or in CA-MHB-Tris plus 44 mM NaHCO_3_ (open symbols) with no drug (circles), 8 μg/ml cefazolin (triangles), 32 μg/ml cefazolin (diamonds), or 15 μg/ml oxacillin (squares) is indicated. The data are means of two independent runs performed in triplicate for each condition ± the SD. Statistical comparisons were made using a Kruskal-Wallis single-factor ANOVA and the *post hoc* pairwise Mann-Whitney U test. Asterisks represent comparisons (**, *P* < 0.01) at the 24-h time point of MRSA 11/11 exposed to 8 μg/ml cefazolin in CA-MHB-Tris versus CA-MHB-Tris plus 44 mM NaHCO_3_ and 15 μg/ml oxacillin in CA-MHB-Tris plus 2% NaCl versus CA-MHB-Tris plus 2% NaCl plus 44 mM NaHCO_3_.

To further quantify the effect of NaHCO_3_ on MRSA β-lactam susceptibility profiles, a time-kill assay was conducted using log-phase responsive MRSA 11/11 or nonresponsive COL cells. As predicted by the MIC data, MRSA 11/11 displayed significantly greater killing when exposed to cefazolin and oxacillin in NaHCO_3_-containing media compared to NaHCO_3_-free media (Fig. 1A). After a 24 h of incubation, a ≥4-log_10_ CFU/ml reduction in counts was observed for MRSA 11/11 exposed to 8 μg/ml cefazolin or 15 μg/ml oxacillin in NaHCO_3_-containing versus NaHCO_3_-free media. These latter β-lactam concentrations represent sublethal concentrations as determined in multiple pilot time-kill studies carried out in NaHCO_3_-free media. In contrast, strain COL displayed a minimal reduction in counts when grown in NaHCO_3_-containing versus NaHCO_3_-free media under all testing conditions (Fig. 1B). Similar differential time-kill results were obtained for the NaHCO_3_-responsive strain MW2 and the NaHCO_3_-nonresponsive strain BMC1001 (Fig. S1). Interestingly, the nonresponsive strain 300-111, which has a relatively low baseline level of resistance to cefazolin and oxacillin, displayed an intermediate level of killing when exposed to 8 μg/ml cefazolin or 15 μg/ml oxacillin in NaHCO_3_-containing versus NaHCO_3_-free media (Fig. S1).

Supplementation with 44 mM NaHCO_3_ had only a modest effect on 24-h growth kinetics for the five study strains (Fig. S2). With the exception of COL and 300-111, stationary-phase cell counts at 24 h were not significantly different between media with versus without 44 mM NaHCO_3_ for any strain tested.

### Population analyses in NaHCO_3_-containing media.

Population analysis profiles (PAPs) are a standard quantitative *in vitro* assessment of the proportions of antibiotic-resistant subpopulations within a given strain versus specific antibiotics (28). MRSA strains contain a variable proportion of highly β-lactam-resistant subpopulations. For example, homogeneously resistant (homoresistant) strains usually contain a high percentage (e.g., >10%) of such subpopulations, whereas heterogeneously resistant (heteroresistant) strains generally contain a lower percentage (e.g., <0.01%) of resistant subpopulations.

**FIG 2:**
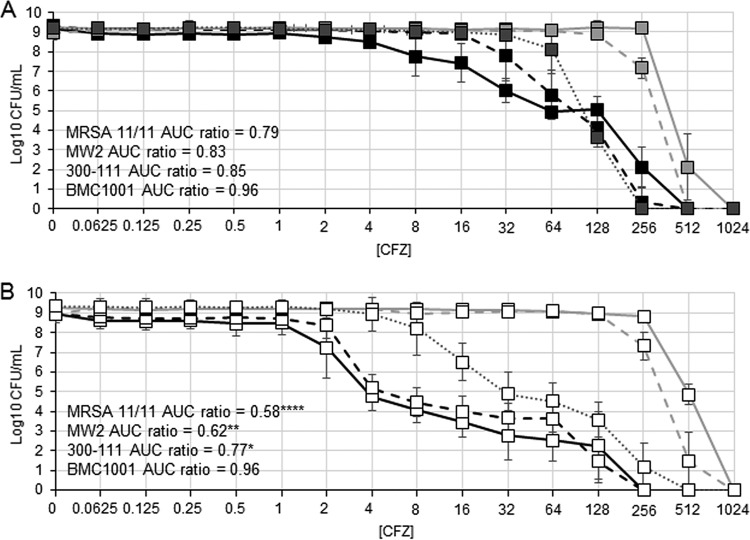
Population analysis of cells grown in media with or without NaHCO_3_. (A) Mueller-Hinton agar (MHA) supplemented with Tris (closed symbols); (B) MHA supplemented with Tris and 44 mM NaHCO_3_ (open symbols). Data for strains MRSA 11/11 (solid black line), MW2 (dashed black line), 300-111 (dotted gray line), COL (solid gray line), and BMC1001 (dashed gray line) are shown. CFZ, cefazolin. Area under the curve (AUC) ratios were compared to COL as a homoresistant reference strain. The data are means of two independent runs performed in triplicate for each condition ± the SD. Statistical comparisons were made using a Kruskal-Wallis single-factor ANOVA and the *post hoc* pairwise Mann-Whitney U test. MRSA 11/11, MW2, and 300-111 AUC in media containing 44 mM NaHCO_3_ were significantly reduced compared to strains in media without NaHCO_3_ (*, *P* < 0.05; **, *P* < 0.01; ****, *P* < 0.0001); the AUC ratio of 300-111 in media containing 44 mM NaHCO_3_ is significantly greater than that of MRSA 11/11 and MW2 (*, *P* < 0.05).

To determine the effect of NaHCO_3_ on β-lactam-resistant subpopulations, PAPs for cefazolin were determined for our five prototype MRSA strains in NaHCO_3_-containing versus NaHCO_3_-free agar. NaHCO_3_-responsive strains MRSA 11/11 and MW2 displayed heteroresistant PAP phenotypes on NaHCO_3_-free agar, whereas NaHCO_3_-nonresponsive strains COL, BMC1001, and 300-111 each displayed a more homoresistant PAP phenotype (Fig. 2A). To better characterize β-lactam heteroresistant versus homoresistant phenotypes, using COL as our benchmark homoresistant strain, we calculated the area under the PAP curve (AUC) ratios for MRSA 11/11, MW2, BMC1001, and 300-111 with respect to the COL AUC. AUC ratios of NaHCO_3_-responsive strains were significantly lower in the presence of NaHCO_3_ (compare Fig. 2A to B). Although NaHCO_3_ did have a slight repressive effect on the resistant subpopulation of 300-111, the AUC for this strain in 44 mM NaHCO_3_ was significantly greater than both MRSA 11/11 and MW2. NaHCO_3_ had no effect on the AUC ratio of BMC1001 or the proportion of highly resistant COL or BMC1001 cells (Fig. 2B). Exposure to oxacillin, with or without NaHCO_3_, yielded similar results to cefazolin, although the magnitude of suppression of the highly resistant subpopulations of the two NaHCO_3_-responsive strains (MRSA 11/11 and MW2) was less than that seen with cefazolin, and the differences were not statistically significant (data not shown).

### Rabbit IE treatment outcomes with β-lactams.

**FIG 3:**
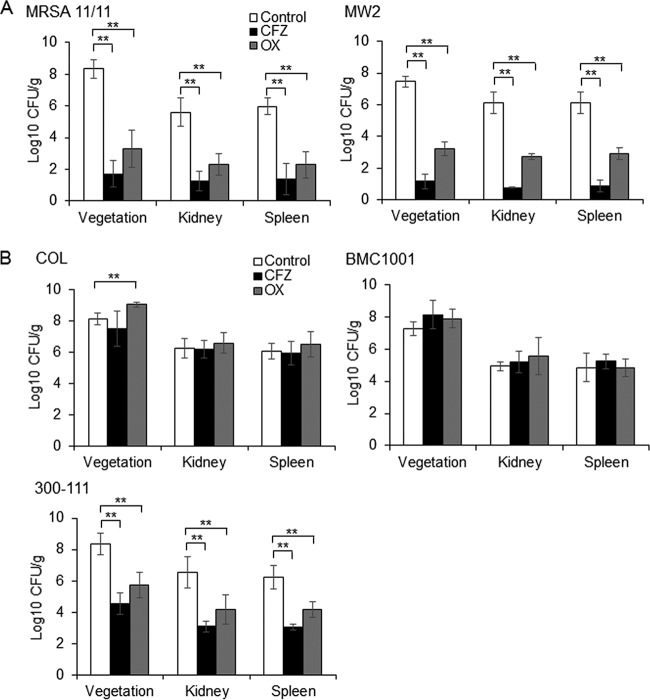
Treatment outcomes of rabbits with infective endocarditis treated with β-lactams. (A) NaHCO_3_-responsive strains; (B) NaHCO_3_-nonresponsive strains. All rabbits were treated with 100 mg/kg of cefazolin (CFZ) or oxacillin (OX), t.i.d., intramuscularly, for 4 days. Data for strains MRSA 11/11 (control, *n* = 6; CFZ, *n* = 8; OX, *n* = 6), MW2 (control, *n* = 7; CFZ, *n* = 6; OX, *n* = 7), COL (control, *n* = 6; CFZ, *n* = 7; OX, *n* = 6), BMC1001 (control, *n* = 6; CFZ, *n* = 7; OX, *n* = 7), and 300-111 (control, *n* = 10; CFZ, *n* = 4; OX, *n* = 6) are shown. The data presented indicate the mean tissue CFU/g for each treatment group ± the SD. Statistical comparisons were made using a Kruskal-Wallis single-factor ANOVA and the *post hoc* pairwise Mann-Whitney U test (**, *P* < 0.01). Strain 300-111 had significantly greater cell counts in all target tissues following cefazolin and oxacillin therapy compared to both MRSA 11/11 and MW2 (*, *P* < 0.05).

To verify the *in vivo* translatability of the NaHCO_3_ responsivity phenotypes determined *in vitro*, a rabbit model of aortic valve IE, treated with the two study β-lactams, was used. The β-lactam treatment regimens were based on (i) published protocols for experimental IE therapy that are capable of clearing MSSA strains from target tissues (5, 29), (ii) human-mimicking pharmacokinetic profiles (5, 29) (Table S2), and (iii) our own pilot treatment outcome studies of experimental MSSA IE with these regimens (Fig. S4). As predicted by *in vitro* MICs determined in NaHCO_3_-containing media, both NaHCO_3_-responsive strains were highly susceptible to β-lactam therapy *in vivo* to levels not dissimilar from that seen with the MSSA control strain ATCC 25923 (Fig. 3A; see also Fig. S4 in the supplemental material). Thus, significant clearance of MRSA 11/11 and MW2 was observed in all target tissues sampled after 4 days of either cefazolin or oxacillin therapy. Of note, sterilization of multiple target tissues was seen in >70% of organ cultures from animals infected with the two NaHCO_3_-responsive when treated with cefazolin; in contrast, oxacillin therapy did not sterilize any target tissues despite significant reductions in MRSA counts in IE caused by these latter strains (data not shown). In contrast, β-lactam therapy was ineffective in reducing bacterial counts in the target tissues of rabbits infected with two NaHCO_3_-nonresponsive strains, COL or BMC1001 (Fig. 3B). The *in vitro* NaHCO_3_-nonresponsive strain, 300-111, displayed a significant reduction in bacterial counts in all target tissues following cefazolin and oxacillin treatment; however, the magnitude of killing was significantly less than that observed in either NaHCO_3_-responsive strain (Fig. 3B). Interestingly, the MICs determined in CA-MHB-Tris containing 44 mM NaHCO_3_ were better predictors of treatment outcomes for all five strains than were the MICs determined in RPMI 1640, based roughly on previously established 2014 CLSI breakpoints (cefazolin breakpoints are no longer employed in the current 2018 CLSI guidelines [13, 30]: S ≤ 8 μg/ml, I = 16 μg/ml, R ≥ 32 μg/ml) and current oxacillin breakpoints (S ≤ 2 μg/ml, R ≥ 4 μg/ml [31]).

It was important to ensure that β-lactam therapy in experimental IE caused by the NaHCO_3_-responsive strains did not select for highly β-lactam-resistant subpopulations within target tissues after β-lactam therapy. Thus, at the time of sacrifice, homogenates from these organs were parallel plated onto tryptic soy agar (TSA) containing 64 μg/ml of either cefazolin or oxacillin in animals treated with these respective agents. No such high-level β-lactam-resistant colonies were detected for either MRSA 11/11-infected or MW2-infected animals after therapy with either of the two β-lactam agents (data not shown).

To investigate whether exposure to NaHCO_3_ itself might influence the tissue burdens of NaHCO_3_-responsive strains during induction of infection, in a separate study, MRSA 11/11 was grown overnight in CA-MHB-Tris containing 25 or 44 mM NaHCO_3_ prior to infection of aortic valve-catheterized animals. At 24 h postinfection, there were no significant differences in target tissue MRSA counts between rabbits infected with MRSA 11/11 grown in NaHCO_3_-free CA-MHB-Tris versus 25 or 44 mM NaHCO_3_-containing CA-MHB-Tris (Fig. S3B). This confirmed that bicarbonate preexposure itself did not hinder the induction and early progression phases of infection in experimental IE caused by NaHCO_3_-responsive strains.

### NaHCO_3_ levels in experimental IE.

To put our *in vivo* outcomes in experimental IE into perspective, we measured [HCO_3_^–^] levels in both infected and uninfected rabbits. Quantification of [HCO_3_^–^] concentrations in the blood of animals with IE versus uninfected controls revealed that blood [HCO_3_^–^] levels remained relatively constant in the range of 20 to 25 mM (Fig. S3C). The fact that the maximal *in vitro* impact of NaHCO_3_ supplementation of standard media was seen at 44 mM versus 25 mM suggested that other factors are likely in play *in vivo* which contribute to NaHCO_3_ responsivity in experimental IE (e.g., host immune molecules and/or cells).

### LL-37 synergy with β-lactams in the presence of physiological concentrations of NaHCO_3_.

We hypothesized that the high-level killing exhibited by β-lactams *in vivo* against the two NaHCO_3_-responsive strains in the presence of physiologic concentrations of NaHCO_3_ (ca. 20 to 25 mM) may be due to a synergistic effect between β-lactams and host immune factors, particularly host defense peptides. To further investigate this, we performed a cell survival assay utilizing sublethal concentrations of the human cathelicidin, LL-37, in combination with cefazolin or oxacillin, in a minimal medium containing 25 mM NaHCO_3_. Strains were exposed to LL-37 and the β-lactams alone or in combination in the presence of this physiological concentration of NaHCO_3._

**FIG 4:**
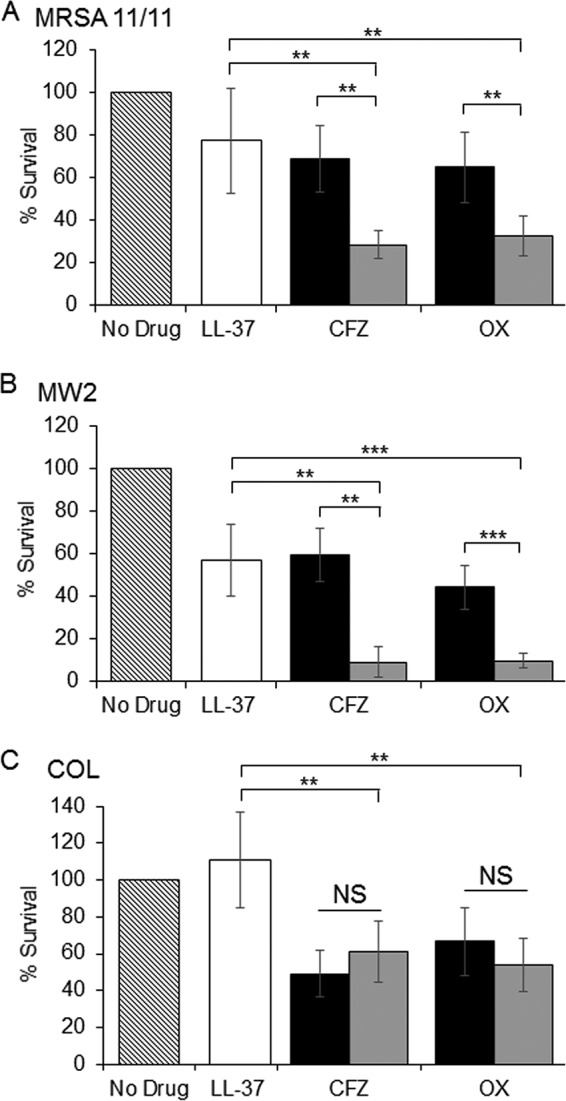
LL-37 synergy with β-lactams in media containing 25 mM NaHCO_3_. (A) MRSA 11/11 (NaHCO_3_ responder); (B) MW2 (NaHCO_3_ responder); (C) COL (NaHCO_3_ nonresponder). CFZ, cefazolin; OX, oxacillin. Bars: no drug, hatched; LL-37 only, white; CFZ or OX, black; CFZ or OX plus LL-37, gray. MRSA 11/11 was exposed to 2.5 μg/ml LL-37 and 0.03125 μg/ml CFZ or OX. MW2 and COL were exposed to 5 μg/ml LL-37 and 0.0625 μg/ml CFZ or OX. The percent survival was calculated after 4 h of exposure to antimicrobials in NaHCO_3_-free DMEM supplemented with 25 mM NaHCO_3_. The data are means of three independent runs performed in triplicate for each condition ± the SD. Statistical comparisons were made using a Kruskal-Wallis single-factor ANOVA and the *post hoc* pairwise Mann-Whitney U test (**, *P* < 0.01; ***, *P* < 0.001).

When exposed to a combination of LL-37 and either cefazolin or oxacillin, both NaHCO_3_-responsive strains displayed significantly lower MRSA survivals compared to either β-lactam agent alone (Fig. 4A and B). In contrast, the NaHCO_3_-nonresponsive strain, COL, did not display synergistic killing when exposed to a combination of LL-37 plus either cefazolin or oxacillin (Fig. 4C), consistent with the results seen in the IE model. These data indicate that, although higher concentrations of NaHCO_3_ (44 mM) are required *in vitro* to disclose “responsiveness” among MRSA strains, host defense and/or other serum factors may foster such responsivity *in vivo* in combination with more physiologic NaHCO_3_ levels (ca. 20 to 25 mM).

### Mechanisms of NaHCO_3_-mediated β-lactam resensitization of MRSA: *mecA* and *sarA* expression.

**FIG 5:**
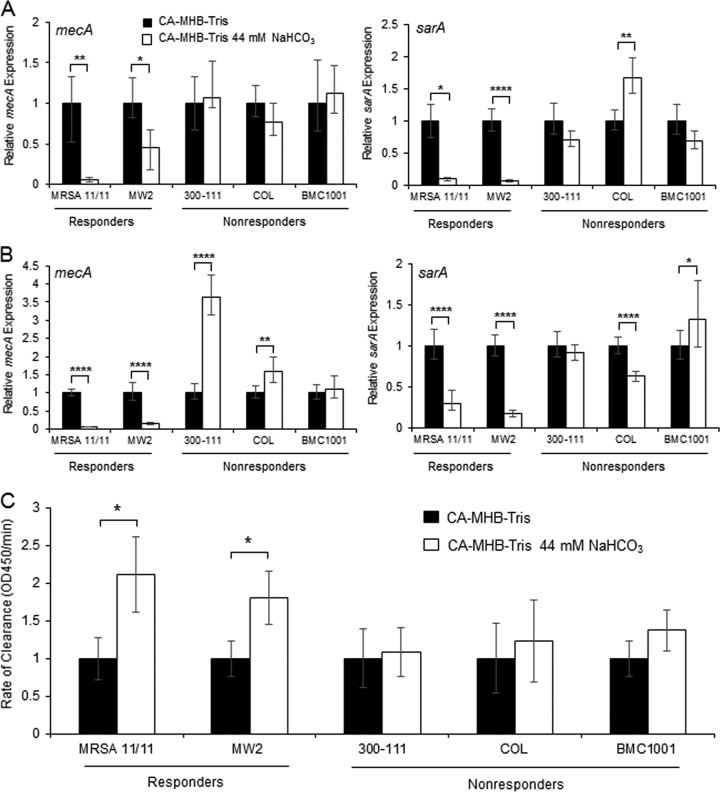
*mecA* and *sarA* expression in NaHCO_3_-responsive and NaHCO_3_-nonresponsive strains. (A) qRT-PCR analysis of *mecA* and *sarA* gene expression in stationary-phase cells grown in CA-MHB-Tris with or without NaHCO_3_. (B) qRT-PCR analysis of *mecA* and *sarA* gene expression in stationary-phase cells grown in CA-MHB-Tris plus 2% NaCl with or without NaHCO_3_ with 1/2 MIC of oxacillin. Gene expression levels are normalized to the housekeeping gene *gyrB*. Gene expression values are normalized to CA-MHB-Tris for each strain. All data are derived from two independent biological replicates, tested in triplicate on two separate occasions. (B) Lipase production analysis in NaHCO_3_-responsive and -nonresponsive strains grown in media with or without NaHCO_3_. Values normalized to CA-MHB-Tris for each strain. The data are means of two independent runs performed in triplicate for each condition ± the SD. Statistical comparisons were made using a Kruskal-Wallis single-factor ANOVA and the *post hoc* pairwise Mann-Whitney U test (*, *P* < 0.05; **, *P* < 0.01; ****, *P* < 0.0001).

To understand the potential genetic basis for altered β-lactam susceptibility in selected MRSA strains in NaHCO_3_-containing media, we investigated the influence of NaHCO_3_ on *mecA* and *sarA* gene expression. The *mecA* locus encodes penicillin-binding protein 2a (PBP2a) that confers β-lactam resistance in MRSA strains (24). In addition, recent studies have demonstrated that the global virulence gene regulator, *sarA*, can also modulate β-lactam resistance via both *mecA*-dependent and *mecA*-independent mechanisms (23). To investigate the influence of NaHCO_3_ exposure on *mecA* and *sarA* gene expression, RNA was extracted from cells grown in media with and without NaHCO_3_ in the absence or presence of 1/2 MIC of oxacillin (to maximally induce *mecA* expression). The quantitative real-time PCR (qRT-PCR) analyses revealed that *mecA* and *sarA* gene expression were each significantly repressed in the two NaHCO_3_-responsive strains under both these *mecA*-noninducing and -inducing conditions (Fig. 5A and B). In contrast, *mecA* gene expression was not repressible in any NaHCO_3_-nonresponsive strain (Fig. 5A and B). The expression of *sarA* was only slightly repressed in COL in NaHCO_3_-supplemented media with oxacillin induction but was nonrepressible in media without oxacillin induction (Fig. 5A and B). The expression of *sarA* was also not repressible in the other NaHCO_3_-nonresponsive strains, BMC1001 and 300-111 (Fig. 5A and B).

To phenotypically verify that the *mecA* repression observed in NaHCO_3_-responsive strains correlated with diminished PBP2a protein production, a macroagglutination PBP2a production assay was utilized. A clear reduction in PBP2a agglutination was observed in both NaHCO_3_-responsive strains after growth in NaHCO_3_-containing media (Table 2; see Fig. S3 in the supplemental material), consistent with the reduced expression of *mecA*. The NaHCO_3_-nonresponsive strains, COL and BMC1001, displayed high levels of agglutination in media with and without NaHCO_3_ exposures (Table 2; see Fig. S3 in the supplemental material). Although *mecA* gene expression was not repressible in strain 300-111 in the presence NaHCO_3_, the level of observable PBP2a agglutination was slightly diminished in media containing NaHCO_3_.

**TABLE 2:** PBP2a agglutination of NaHCO_3_-responsive and -nonresponsive strains grown in media with or without 44 mM NaHCO_3_

Strain	PBP2a agglutination level^*a*^	
	CA-MHB-Tris	CA-MHB-Tris, 44 mM NaHCO_3_
MRSA 11/11	++	–
MW2	++	–
COL	+++	+++
BMC1001	+++	+++
300-111	+++	++

a Interpretation of agglutination intensity: +++, high; ++, moderate; +, low; and –, none.

Lipase production is one of the signature phenotypes normally repressed by the global regulator *sarA* (32, 33). To phenotypically confirm the blunting of *sarA* gene expression by NaHCO_3_ exposures seen in NaHCO_3_-responsive versus -nonresponsive strains, a tributyrin clearance assay was used to measure lipase production. As predicted by NaHCO_3_ repression of *sarA* gene expression, NaHCO_3_-responsive strains had significantly higher levels of lipase activity when exposed to NaHCO_3_ (Fig. 5C). In contrast, lipase production in NaHCO_3_-nonresponsive strains was not affected by the presence of NaHCO_3_ (Fig. 5C).

## DISCUSSION

Several studies have demonstrated the importance of NaHCO_3_, the body’s primary biological buffer, in altering susceptibility of S. aureus to various antibiotics, including β-lactams, as well as to the prototypical host defense peptide, LL-37 (17, 21, 27). Here, we show the ability of NaHCO_3_ to increase susceptibility in selected MRSA strains to two conventional β-lactam agents commonly used to treat MSSA infections. This raises the intriguing possibility of treating MRSA infections with such β-lactam agents, a concept not currently endorsed by any therapeutic guidelines for MRSA (22). Since such β-lactams are relatively inexpensive and exhibit infrequent serious side effects (34), our findings are of potential clinical relevance. The results show that certain MRSA strains found to be β-lactam susceptible *in vitro* by AST with NaHCO_3_ supplementation can be effectively treated by β-lactam therapy in a prototypical model of endovascular infections, experimental IE.

*In vitro*, NaHCO_3_-responsive MRSA strains have a typical signature. They are heteroresistant on PAPs and have relatively low MICs within the MRSA range to conventional β-lactams (i.e., to cefazolin and oxacillin). Phenotypically, compared to NaHCO_3_-nonresponsive MRSA strains, such NaHCO_3_-responsive MRSA strains are killed significantly better *in vitro* by these β-lactams in the presence versus absence of NaHCO_3_, and NaHCO_3_ substantially represses the growth of their resistant subpopulations.

Importantly, not all strains with relatively low intrinsic β-lactam MICs display the NaHCO_3_-responsive phenotype, as demonstrated by strain 300-111. Despite displaying nearly identical basal β-lactam MICs to MRSA 11/11 in standard media, this “intermediate” NaHCO_3_-nonresponsive strain was incompletely cleared from target tissues in experimental IE following cefazolin and oxacillin treatment. These outcomes underscore the concept that the resistant subpopulations of such low-MIC, but NaHCO_3_-nonresponsive strains cannot be simply eliminated by achieving supra-MIC β-lactam serum levels in such infections. More such “intermediate” strains need to be studied in the IE model to verify this concept.

In our investigations, AST was performed at both 44 mM NaHCO_3_, a concentration present in certain cell culture media (e.g., DMEM), as well as at 25 mM NaHCO_3_, a physiologically relevant concentration for humans and rabbits (35). Although we saw highly effective β-lactam-mediated killing of our “responsive” strains *in vivo*, we saw only a modest impact at physiologically relevant concentrations of 25 mM NaHCO_3_ *in vitro*. These results suggested that other factors previously implicated in endovascular pathogenesis, such as host defense peptides (e.g., LL-37 or α-defensin, hNP-1 [36–38]), might synergize with β-lactams at physiological NaHCO_3_ concentrations to yield *in vivo* killing mirroring those observed *in vitro* at 44 mM NaHCO_3_. Of note, data, at least for LL-37, supported this notion. We recognize that other innate host immune factors (e.g., neutrophils, serum complement, other host peptides, antistaphylococcal antibodies, etc.) may also contribute to enhanced bacterial killing by β-lactams *in vivo* in the presence of physiological concentrations of NaHCO_3_.

In terms of how physiological concentrations of NaHCO_3_ might impact MRSA to alter its intrinsic β-lactam susceptibility profiles, Farha et al. (27) proposed that this molecule was simply acting through its capacity to collapse the proton motive force (PMF; PMF = Δψ + ΔpH). Thus, [HCO_3_^–^], via Le Chatelier’s principle, would drive the reaction, H^+^ + [HCO_3_^–^] >>>> H_2_O + CO_2_ to consume protons and collapse PMF by mitigating the ΔpH. In their study, these researchers found that an MSSA strain became more resistant to oxacillin in the presence of NaHCO_3_ (27) and attributed their observations to globally decreased cellular respiration and growth rates. Since β-lactams are more effective against rapidly dividing cells, these authors postulated the mechanism to be based in an overall impact of reduced respiratory energy production to fuel growth in the presence of PMF-modifying concentrations of NaHCO_3_. In contradistinction, our study observed essentially the opposite result in MRSA, i.e., that certain MRSA strains become highly susceptible to β-lactams in the presence of NaHCO_3_, indicating that additional changes to MRSA-defining gene expression patterns may overcome any inhibitory effect of NaHCO_3_ on global cell metabolism to render such strains as β-lactam susceptible.

The β-lactam treatment of rabbits infected with *in vitro* NaHCO_3_-responsive strains was highly effective in clearing infection in all target organs assessed. It was hypothesized that a decrease in overall virulence stimulated by growth in a NaHCO_3_-containing microenvironment might be part of the explanation for increased susceptibility to β-lactam antibiotics under these conditions. However, overnight growth of the NaHCO_3_-responsive strain, MRSA 11/11, in the presence of 25 or 44 mM NaHCO_3_ prior to infection had no effect on the strain’s ability to induce or propagate experimental IE.

Intriguingly, our *in vitro* MIC data obtained in CA-MHB containing 44 mM NaHCO_3_ was a better predictor of *in vivo* outcomes than the tissue culture medium RPMI 1640. Although use of the latter medium for AST is gaining some traction as a “host mimicking milieu” (especially for Gram-negative pathogens [20, 25, 39]), we found that it falsely predicted the efficacy of oxacillin and cefazolin for the *in vivo* treatment of some study MRSA strains, (e.g., BMC1001). Further head-to-head screening in both media, using larger MRSA strain collections, will be required to fully identify which medium is the best predictor of β-lactam efficacy *in vivo* against MRSA.

Gene expression analyses revealed that *mecA* and *sarA* were highly repressed *in vitro* in NaHCO_3_-responsive versus NaHCO_3_-nonresponsive strains. Repression of the *mecA* gene was, in turn, found to directly correspond to a decrease in PBP2a protein expression. It is unclear, however, whether NaHCO_3_ has a direct impact on *mecA* expression, or whether altered *mecA* gene expression is mediated through repression of *sarA* or other global regulons. Recent studies have shown that deletion of *sarA* and *sigB* can reduce *mecA* expression (23); therefore, NaHCO_3_ may be altering *mecA* expression directly and/or through one or both of these global regulatory pathways. In addition, we observed that the *mecA* and *sarA* expression levels were reduced by NaHCO_3_ exposures in both the presence and the absence of oxacillin induction (Fig. 5A and B). This raises an intriguing dual-mechanism notion that NaHCO_3_ can directly effect the basal expression of these genes, as well as interfere with *mecA* induction by β-lactams, such as oxacillin.

As discussed above, Dorschner et al. determined that NaHCO_3_ had a significant repressive impact on expression of the E. coli homologue of the key S. aureus stress-response regulon, *sigB* (21). This locus is known to regulate pigment production in S. aureus (40); thus, factors that repress *sigB* expression ultimately reduce carotenoid production, altering membrane fluidity (41). Carotenoid is critical for oligomerization and proper insertion of PBP2a into the cell membrane (42). NaHCO_3_’s potentially repressive impact on the *sigB* regulator*y* axis could thus result in a decrease in carotenoid production. This event, combined with decreased PBP2a expression in NaHCO_3_-responsive strains, could yield MRSA cells that are phenotypically “*mecA* defective.” In this regard, pilot studies in our laboratory have confirmed that *in vitro* NaHCO_3_ exposures in both “responsive” and “nonresponsive” MRSA can repress *sigB* expression, as well as carotenoid production (data not shown). Thus, bicarbonate repression of the *sigB* regulator*y* axis does not appear to play a principal role in β-lactam resensitization by bicarbonate in MRSA.

Although we have identified at least two genetic targets of NaHCO_3_ that may influence MRSA resistance to β-lactams, it is highly likely that NaHCO_3_ has pleotropic effects on multiple gene expression profiles which may contribute to β-lactam susceptibility in NaHCO_3_-responsive strains. Furthermore, different pathways may be activated or repressed in individual “responsive” and “nonresponsive” strains, resulting in multiple “genetic types” of NaHCO_3_ responsiveness. For example, wall teichoic acid (WTA) forms a scaffold for PBP2a maturation, allowing its insertion into the cell membrane (43). Deletion of genes involved in WTA synthesis can render MRSA strains more susceptible to β-lactams (43, 44), indicating that this may be another potential site of NaHCO_3_ action on gene expression. The modest decrease in PBP2a expression in strain 300-111, despite *mecA* expression being nonrepressible by NaHCO_3_ in this strain, highlights the latter point. Thus, NaHCO_3_ may be affecting multiple pathways in this nonresponsive strain, causing an increase in *mecA* gene expression but a slight overall decrease in membrane insertion of mature PBP2a.

Another potential “checkpoint” for NaHCO_3_ is PBP4, a protein involved in the generation of highly cross-linked peptidoglycan. Although this PBP is normally dispensable in MSSA (45, 46), MRSA strains require its activity for proper peptidoglycan cross-linking (47), without which it is dependent on the β-lactam-susceptible PBP2 for peptidoglycan synthesis. Interestingly, inhibition of PBP4 activity has been shown to diminish cell wall cross-linking in β-lactam heteroresistant MRSA strains but had no effect on cross-linking in homoresistant strains (48). Currently, little is known about the regulation of PBP4 expression (49); however, further investigations into the effect of NaHCO_3_ on PBP4 protein production may offer additional insights into mechanisms underlying NaHCO_3_-responsive versus NaHCO_3_-nonresponsive phenotypes.

Other potential genes that might be involved in the “bicarbonate effect” in MRSA include *prsA*, *vraSR*, *mprF*, and *graSR* (50–57). Each of these genes has been implicated in a phenomenon somewhat akin to NaHCO_3_/β-lactam responsivity called the “seesaw” effect, in which MRSA cells that evolve daptomycin resistance become resensitized to β-lactams (50–53). Many of these genetic perturbation possibilities are under active investigation in our labs using whole-genome sequencing and RNA sequencing analyses.

One of the major limitations of our study is the fact that only five MRSA strains were investigated. We are currently screening a large collection of well-characterized clinical MRSA strains which represent the broad range of clonal complex, *agr*, SCC*mec*, and *spa* types in current worldwide circulation for their NaHCO_3_-responsive profiles *in vitro*. Subsets of these strains will then be subjected to the same *in vivo* testing in the IE model as in the current work to further verify the linkage between *in vitro* NaHCO_3_ responsivity and effective β-lactam therapy *in vivo*.

A key *in vivo* finding in the present study was that, in NaHCO_3_-responsive MRSA IE, neither treatment with oxacillin nor treatment with cefazolin selected for the emergence of high-level (MICs > 64 μg/ml) β-lactam-resistant subpopulations within cardiac vegetations. It should be emphasized, however, that one additional limitation of the present investigation was that rabbit vegetations are considerably smaller than those of humans with IE (∼2 to 3 mm in diameter [corresponding to a weight of ∼50 to 100 mg] versus ∼1 cm in diameter [corresponding to ∼500 to 1,000 mg], respectively) (50–52). Therefore, if bacterial densities observed in the rabbit IE model (which can reach 10^8^ to 10^9^ CFU/gm) are similar to those in humans, then the total bacterial burden in human vegetations would be at least 10 times higher than in the rabbit IE model. This would correspondingly increase the chance that highly resistant subpopulations might well emerge during β-lactam therapy in human IE. This metric will need to be carefully monitored in any future clinical trials. Once a reliable and facile method for identifying potentially β-lactam-responsive MRSA strains has been established and verified, large-scale clinical trials to evaluate the effectiveness of β-lactam therapy for treating selected MRSA infections in human patients would be warranted.

## MATERIALS AND METHODS

### Bacterial strains and media.

The methicillin-resistant Staphylococcus aureus (MRSA) strains used in this study were all initially derived from patients with clinical infections: MRSA 11/11 (USA300), MW2 (USA400), COL (USA100), BMC1001 (USA500), and 300-111 (CC8, *spa* type 4, Iberian clone) (53–58). These prototypical strains encompass the range of clonal complex, *agr*, and SCC*mec* genotypes that are in current worldwide clinical circulation. In addition, several of these strains have been previously used in experimental studies of virulence, pathogenesis and antimicrobial responsiveness (MW2, COL, and MRSA 11/11) (5, 23, 56, 59). In experimental IE studies, we also included the well-known MSSA strain, ATCC 25923, as a control.

MRSA and MSSA strains were stored at –80°C until thawed for use. They were isolated on tryptic soy agar (TSA) and incubated at 37°C in ambient air. Bacteria were grown for most experiments, including AST, overnight in cation-adjusted Mueller-Hinton broth (CA-MHB; Difco) with the addition of 100 mM Tris (hydroxymethyl-aminomethane) to maintain pH at ∼7.3 ± 0.1 throughout all AST testing (Fisher Scientific). In parallel studies, CA-MHB-Tris was supplemented with either 25 or 44 mM NaHCO_3_; these NaHCO_3_ concentrations represent physiologic bloodstream concentrations, and those found in DMEM, respectively. In parallel control assays, AST testing was also performed in the tissue culture medium, Roswell Park Memorial Institute (RPMI) 1640 (Fisher Scientific) supplemented with 10% Luria-Bertani (LB) broth. All media were supplemented with 2% NaCl when assays were performed in which MRSA cells were exposed to oxacillin.

### MIC assays.

The MICs of cefazolin and oxacillin were determined according to the Clinical and Laboratory Standards Institute (CLSI) guidelines by broth microdilution (12, 13). MRSA were grown overnight in specified media and diluted into the same media containing 2-fold serial dilutions of antibiotics. We added 2% NaCl to all media when performing oxacillin MICs. All MIC values are the mode of at least six independent determinations.

For MIC determinations performed in the presence or absence of salicylic acid, we used CA-MHB plus 100 mM Tris (CA-MHB-Tris) with or without 25 or 50 μg/ml salicylic acid.

### Time-kill assays.

Cells were grown overnight in specified testing media and diluted to 5 × 10^5^ CFU/ml in 200 μl of same media on a 96-well plate (flat bottom; tissue culture-treated). Based on pilot experiments, cells were incubated at 37°C for 3 h to enter log phase and then diluted to 5 × 10^5^ CFU/ml in 200 μl of the same media, with or without antibiotic, in a 96-well flat-bottom plate. Plates were then incubated at 37°C for 24 h. Surviving cells were quantified at 0, 2, 4, 8, and 24 h of incubation, and data are expressed as the log_10_ CFU/ml. A bactericidal effect was defined as a ≥3-log_10_ CFU/ml decline in counts at 24 h versus the count at 0 h.

### Population analysis profiles.

The PAP protocol was modified from published guidelines (60–63). In brief, agar plates were prepared with Mueller-Hinton Agar (MHA) supplemented with 100 mM Tris, 2% NaCl, and 44 mM NaHCO_3_ (where indicated) at pH ∼7.3 ± 0.1, containing 2-fold serial dilutions of cefazolin or oxacillin at concentrations ranging from 0.0625 to 1024 μg/ml. Bacterial cells were grown overnight in broth testing medium and diluted to ∼1 × 10^9^ CFU/ml in phosphate-buffered saline (PBS). Ten-fold serial dilutions were performed in PBS, and 10 μl of each dilution was plated onto the MHA plates. After 48 h of incubation at 30°C, plates were enumerated for viable cells at each drug concentration. The area under a population analysis profile (PAP) curve (AUC) was calculated by linear approximation.

### Synergy of β-lactams with host defense peptides.

For studies of β-lactam synergy with host defense peptides, a prototypical host defense peptide, LL-37, was used. This cathelicidin peptide is commonly found in large amounts in human epithelial cells, as well as in neutrophils (64, 65), and has been documented to play an important role in innate immunity (66–68). Highly purified LL-37 was purchased commercially from Peptides International (Lexington, KY).

MRSA strains were grown overnight in NaHCO_3_-free DMEM (Gibco) supplemented with 25 mM NaHCO_3_ and diluted into the same medium supplemented with 150 mM NaCl. Diluted cells were incubated for 3 h at 37°C to enter log-phase growth. Log-phase cells were then diluted to 1 × 10^3^ CFU/ml with LL-37, cefazolin, and oxacillin, alone or in combination; this inoculum has been used as a standard in our prior killing assays with host defense peptides (69). The final concentrations of the antimicrobials were as follows: 2.5 μg/ml LL-37 (MRSA 11/11), 5 μg/ml LL-37 (MW2 and COL), 0.03125 μg/ml cefazolin and oxacillin (MRSA 11/11), and 0.0625 μg/ml cefazolin and oxacillin (MW2 and COL). These antibiotic concentrations were determined after extensive pilot studies to represent individual drug levels that did not cause ≥50% killing of this starting MRSA inoculum. Surviving cells were quantified after 4 h of incubation with antimicrobials at 37°C, and the percent survival was calculated at this time point as follows: (CFU with antimicrobial/CFU with no antimicrobial) × 100.

### Isolation of RNA and quantitative real-time PCR analyses.

To quantify expression of two key genes involved in the MRSA phenotype (*mecA* and *sarA*), total RNA was isolated from the study strains following overnight growth in media with or without NaHCO_3_ supplementation using an RNeasy kit (Qiagen, Valencia, CA) (59). Cells were grown overnight in specified media (CA-MHB-Tris ± 44 mM NaHCO_3_) and then diluted 1:100 into the same media and, followed by incubation at 37°C overnight. To quantify the combined effect of oxacillin stimulation and NaHCO_3_ exposure on gene expression, cells were grown as specified, but diluted into media containing 1/2 MIC of oxacillin (with 2% NaCl). qRT-PCR was performed using primers for *mecA*, *sarA*, and *gyrB* as previously described (59, 70, 71). *gyrB* was used as a housekeeping gene to normalize transcript quantifications. Relative quantification was calculated using the ΔΔ*C_T_* method. All qRT-PCR gene expression data were determined from two separate biological replicates for each condition, tested in triplicate. The data are presented as the fold change in gene expression in the presence of NaHCO_3_ exposures compared to CA-MHB-Tris alone for each strain, with CA-MHB-Tris gene expression data being normalized to 1.0.

### PBP2a agglutination assays.

A semiquantitative, rapid, and reliable latex agglutination method (Seiken, Tokyo, Japan) was used to measure PBP2a production (72), using beads labeled with specific anti-PBP2a antibody (73). Strains were grown overnight in specified media (CA-MHB-Tris ± 44 mM NaHCO_3_) at 37°C. Cells were collected by centrifugation, washed once in PBS, resuspended in PBS to an optical density at 600 nm (OD_600_) of 1.0, and pelleted. Pellets were prepped for PBP2a agglutination according to the manufacturer’s instructions. Agglutination results were scored blindly and separately by two investigators (S.C.E. and L.L.) and scored as high (+++), moderate (++), low (+), or negative (–) based on the presence or absence of an overt agglutination pattern. S. aureus ATCC 43300 (MRSA; PBP2a positive) and ATCC 25923 (MSSA; PBP2a negative) were used as positive and negative controls, respectively, in all assays.

### Lipase assays.

Lipase production is normally repressed by *sarA* (33, 74). As a phenotypic readout for NaHCO_3_-mediated *sarA* repression, a spectrophotometric assay measuring the rate of clearance of a tributyrin emulsion was used to measure lipase activity (75). Strains were grown overnight in specified media (CA-MHB-Tris ± 44 mM NaHCO_3_) at 37°C and then diluted to ∼1 × 10^9^ CFU/ml in same media and filter sterilized. A 0.5% (vol/vol) tributyrin solution (Sigma) was prepared in 100 mM Tris (pH 8.0) plus 25 mM CaCl_2_ and emulsified by sonication for 3 min. The tributyrin emulsion was diluted 1:1 with a 0.8% (wt/vol) low-gelling-temperature agarose (Sigma), and the suspension was maintained at 50°C. Then, 1 ml of the tributyrin suspension was added to 100 μl of each supernatant in a spectrophotometric cuvette, and the OD_450_ was measured at time zero and at 1, 2, 3, 4, and 5 min. Lipase activity was calculated as the rate of clearance normalized to the cell density of each sample. A higher rate of clearance corresponds to greater lipase activity. The data are presented as the fold change in lipase activity in the presence of NaHCO_3_ compared to CA-MHB-Tris alone for each strain, with CA-MHB-Tris alone lipase activity being normalized to 1.0. As stated above, increases in lipase activity correlate with repression of the *sarA* gene “tone” (activity) (33).

### Rabbit model of MRSA infective endocarditis.

To verify the *in vivo* translatability of the relationship between NaHCO_3_ responsiveness or NaHCO_3_ nonresponsiveness observed *in vitro*, a well-characterized rabbit model of indwelling catheter-induced aortic valve infective endocarditis (IE) was used (59). This model is ideal for these studies since it represents a combined acute bacteremia and subacute multitarget organ, high-inoculum infection (66). Rabbits were infected intravenously at 48 h after catheter placement with 2 × 10^5^ CFU/animal of the indicated strain; this inoculum represents the 95% infective dose for inducing IE, as established by extensive pilot experiments for each strain. At 24 h postinfection, the animals were randomized into either an untreated control group (sacrificed at this time point as a therapeutic baseline) or β-lactam-treated groups (100 mg/kg cefazolin or oxacillin, administered by intramuscular injection, three times daily [t.i.d.] for 4 days). These β-lactam treatment strategies encompass (i) dose regimens used in prior studies of experimental IE (5) and (ii) doses that mimic human-like pharmacokinetics in experimental IE (29).

To provide a perspective on the extent of β-lactam-mediated killing *in vivo* in experimental IE among NaHCO_3_-responsive versus NaHCO_3_-nonresponsive MRSA, we performed a parallel study using the highly cefazolin-susceptible MSSA strain, ATCC 25923, treated with the same cefazolin treatment regimen used above for MRSA IE.

In all studies, at 24 h after the last antibiotic treatment, the animals were sacrificed, and their cardiac vegetations, kidneys, and spleens were removed and quantitatively cultured on TSA. MRSA counts were expressed as the mean log_10_ CFU per gram of tissue (± the standard deviations [SD]). To assess the potential emergence of high-level resistance to either cefazolin or oxacillin for strains MRSA 11/11 and MW2 during such β-lactam treatments, the three target tissues were parallel plated on the above media, but containing 64 μg/ml of the antibiotic of interest. The limit of detection in target organ cultures in this model, based on average target tissue weights, is ≤2 log_10_ CFU/g.

To determine whether preincubation of NaHCO_3_-responsive MRSA in media containing NaHCO_3_ itself might influence the initial induction and/or early progression phases of experimental IE, MRSA 11/11 was grown overnight in CA-MHB-Tris with either 25 or 44 mM NaHCO_3_. Rabbits were then infected intravenously at 48 h after catheter placement with 2 × 10^5^ CFU/animal with NaHCO_3_-preexposed cells. At 24 h postinfection, the rabbits were sacrificed, and the same target tissues described above were removed and quantitatively cultured on TSA.

### Statistics.

All statistical comparisons were made using a Kruskal-Wallis single-factor analysis of variance (ANOVA) test and a pairwise Mann-Whitney U test *post hoc* comparison. The data are presented, unless otherwise indicated, as the sample means ± the SD. *P* values of <0.05 were considered statistically significant.

### Study approval.

Female New Zealand White rabbits (weighing 2.2 to 2.5 kg) were used in all animal studies (Irish Farm). The rabbits were maintained in accordance with the American Association for Accreditation of Laboratory Animal Care criteria. The Institutional Animal Care and Use Committee of the Los Angeles Biomedical Research Institute at Harbor–UCLA Medical Center approved all animal study protocols.

## Supplementary Material

Supplemental file 1
