# Supplementary material for: Bicarbonate Resensitization of Methicillin-Resistant Staphylococcus aureus to β-Lactam Antibiotics
Source: Antimicrob Agents Chemother. 2019 Jun 24;63(7):e00496-19. doi: 10.1128/AAC.00496-19 (PMC6591647; doi:10.1128/AAC.00496-19)
Supplement: Supplemental file 1 [file AAC.00496-19-s0001.pdf]

Figure S1

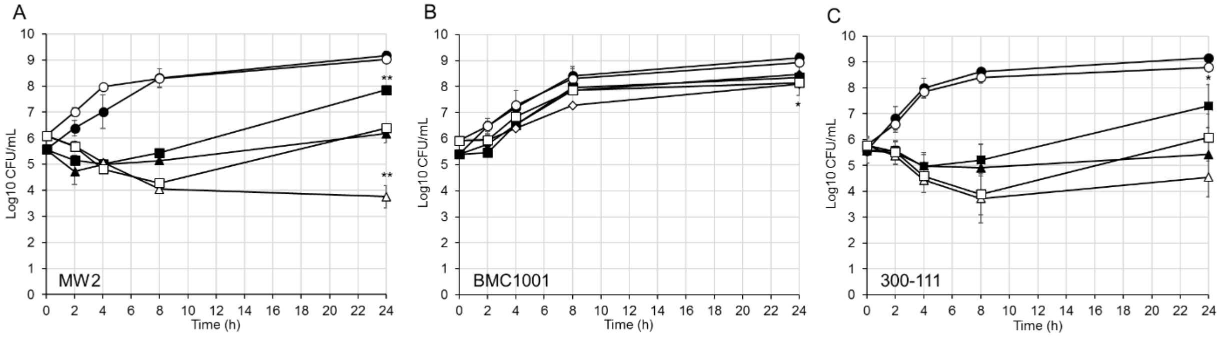

Figure S1. Time kill of log phase cells grown in media with and without $NaHCO_3$. (**A**) MW2 (**B**) BMC1001 (**C**) 300-111. Growth in CA-MHB Tris (closed symbols), growth in CA-MHB Tris 44 mM $NaHCO_3$ (open symbols), no drug (circles), 8 µg/mL cefazolin (triangles), 32 µg/mL cefazolin (diamonds), 15 µg/mL oxacillin (squares). 2% NaCl was supplemented in all media when cells were exposed to oxacillin. The data are the means of two independent runs performed in triplicate for each condition ± the standard deviation. Statistical comparisons were made using a Kruskal-Wallis Single Factor ANOVA and post hoc Pairwise Mann-Whitney *U* test. Asterisks represent comparisons between CA-MHB Tris ± 44 mM $NaHCO_3$ at 24 hour time point for the following drug concentrations: 8 µg/mL cefazolin (MW2, 300-111), 32 µg/mL cefazolin (BMC1001), 15 µg/mL oxacillin (MW2, 300-111), $^*P < 0.05$, $^{**}P < 0.01$.

Figure S2

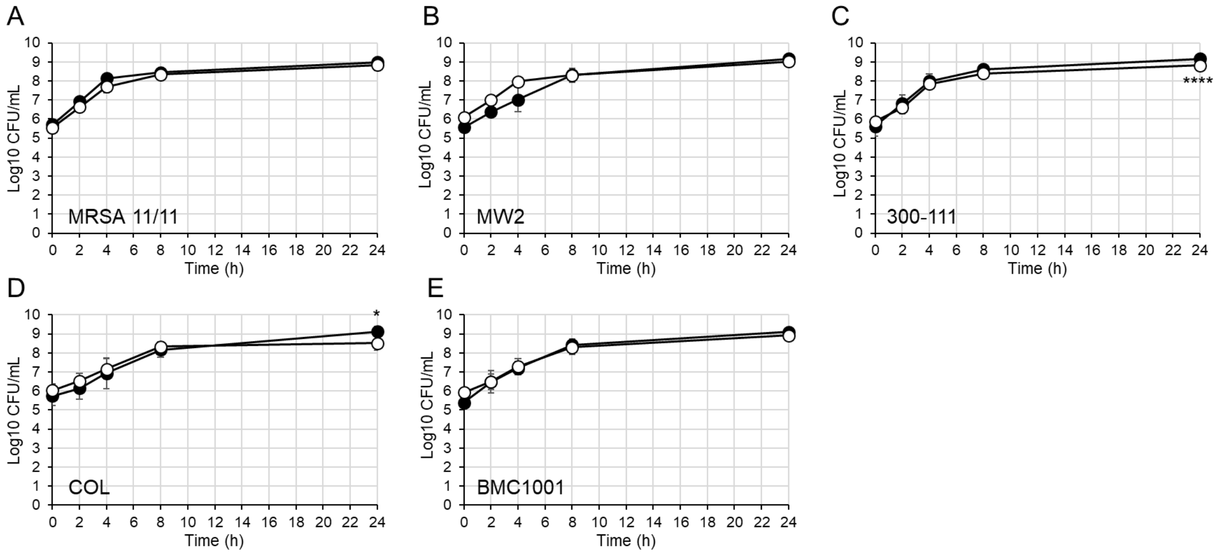

Figure S2. Growth curve kinetics of cells grown in media with and without $NaHCO_3$. (**A**) MRSA 11/11 (**B**) MW2 (**C**) 300-111 (**D**) COL (**E**) BMC1001. Growth in CA-MHB Tris (closed symbols), growth in CA-MHB Tris 44 mM $NaHCO_3$ (open symbols). The data are the means of two independent runs performed in triplicate for each condition ± the standard deviation. Statistical comparisons were made using a Kruskal-Wallis Single Factor ANOVA and post hoc Pairwise Mann-Whitney *U* test at 24 h between cells grown in media with and without 44 mM $NaHCO_3$ for each strain, $^{*}P < 0.05$, $^{****}P < 0.0001$.

Figure S3

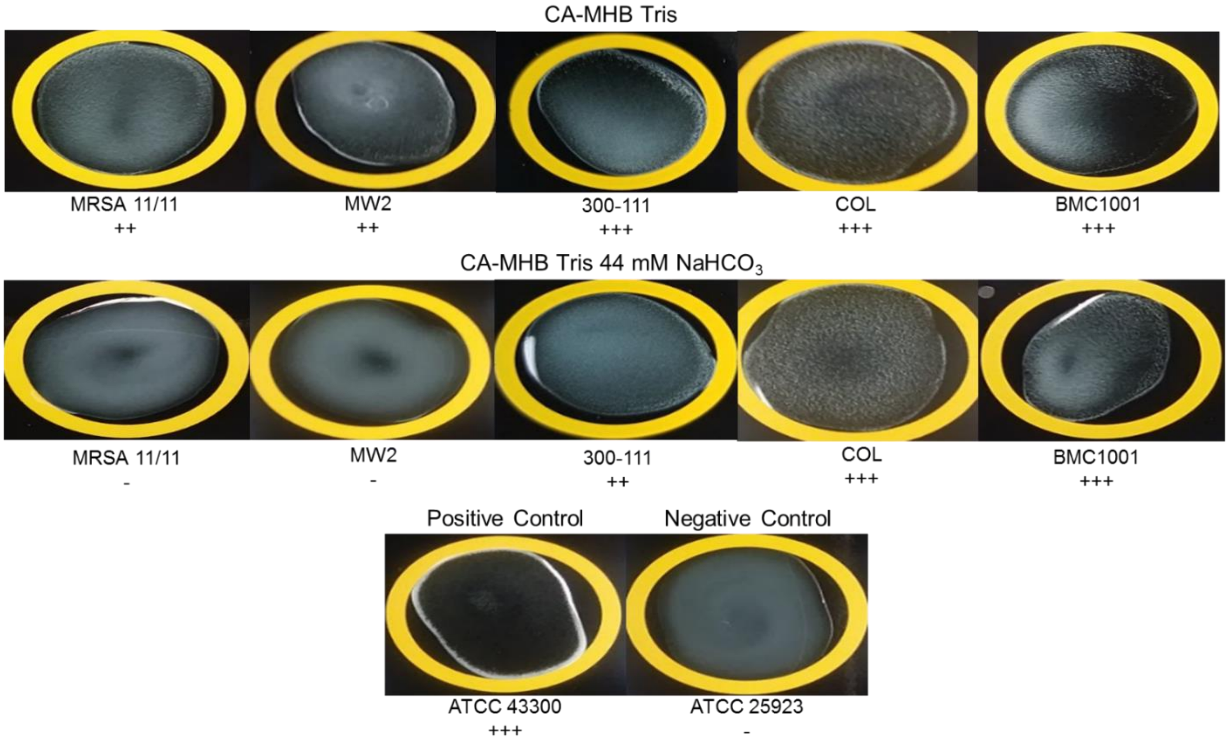

Figure S3. PBP2a agglutination of $NaHCO_3$-responsive and nonresponsive strains. Interpretation of agglutination intensity: +++, high; ++, moderate; +, low; -, none. Strains ATCC43300 and ATCC25923 were used as positive and negative controls, respectively, in all assays. Cells were grown overnight in CA-MHB 100 mM Tris ± 44 mM $NaHCO_3$ (without oxacillin induction). The data are derived from three independent agglutination analyses performed in duplicate, with the scoring and images being the most phenotypically representative of all trial outcomes.

Figure S4

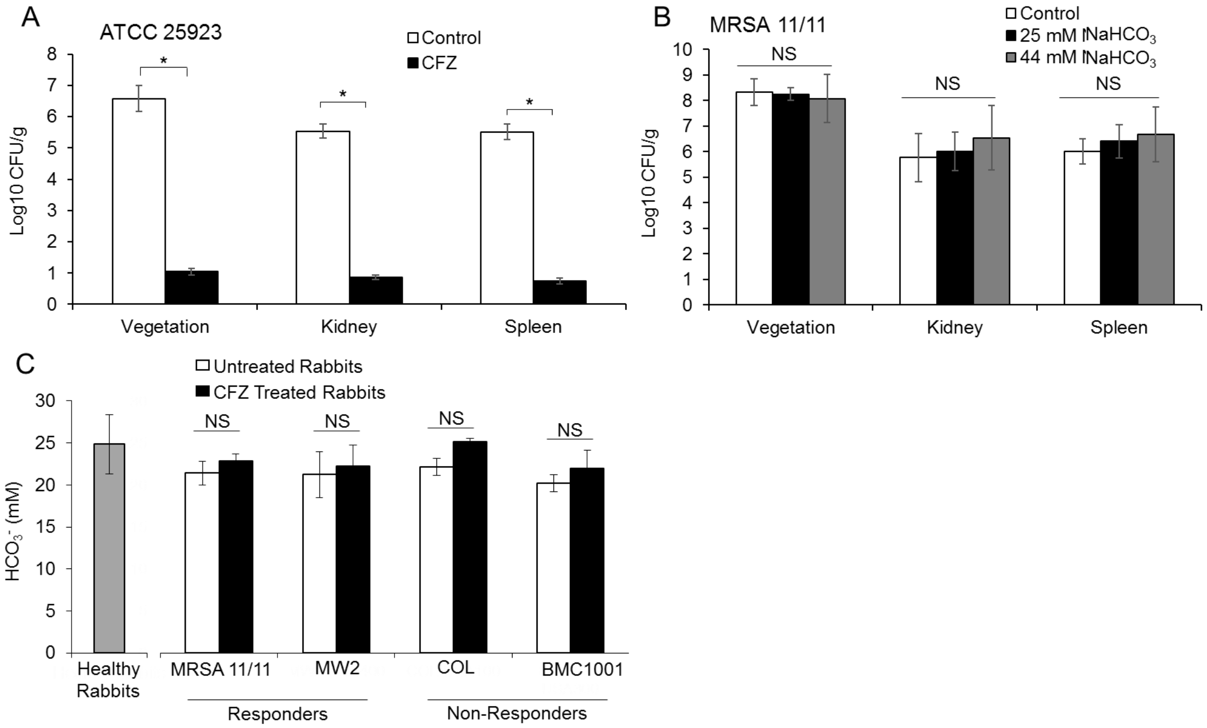

Figure S4. Comparative treatment of experimental IE due to MSSA strain (A); Pathogenicity of MRSA pre-exposed to $NaHCO_3$ in experimental IE (B); and Rabbit serum analysis of HCO_3 levels (C). (A) Treatment outcomes for rabbits infected with MSSA strain ATCC25923 and treated with 100 mg/kg cefazolin (CFZ), t.i.d. for 4 days. **Control** n = 3, **CFZ** n = 5 (**B**) Pathogenicity of MRSA 11/11 grown in $NaHCO_3$-containing media prior to infection. **Control** n = 7, **25 mM $NaHCO_3$** n = 5, **44 mM $NaHCO_3$** n = 5 (**C**) HCO_3^- concentration in rabbit blood before and after infection. **Untreated rabbits** n = 3 for all strains, **CFZ treated rabbits** n = 3 for all strains. The data presented are the mean tissue CFU/g for each treatment group or mean serum [HCO_3^-] ± the standard deviation. Statistical comparisons were made using a Kruskal-Wallis Single Factor ANOVA and post hoc Pairwise Mann-Whitney *U* test, *P < 0.05. No statistical significance was observed for HCO_3^- concentrations in healthy rabbits compared to rabbits infected with any of the four study strains.

Table S1. Minimum Inhibitory Concentration (MIC) of β-lactam antibiotics against methicillin-resistant *Staphylococcus aureus* grown in media with and without salicylic acid (SAL).

	Cefazolin MIC (µg/mL)				Oxacillin MIC (µg/mL)			
	MRSA 11/11	MW2	COL	BMC1001	MRSA 11/11	MW2	COL	BMC1001
Ca-MHB	16	4	256	256	32	32	512	128
Ca-MHB Tris	16	8	256	256	32	64	512	256
Ca-MHB Tris 25 µg/mL SAL	32	16	512	256	32	32	512	256
Ca-MHB Tris 50 µg/mL SAL	32	16	256	256	64	32	512	256

Table S2. Serum concentration of cefazolin and oxacillin in rabbits after a 100 mg/kg i.m. injection.

Drug (Dose)	Serum$_{[Drug]}$ 1 h	2 h
Cefazolin (100 mg/kg)	117.7 µg/mL ± 10.6	38.6 µg/mL ± 5.9
Oxacillin (100 mg/kg)	23.2 µg/mL ± 4.3	5.5 µg/mL ± 1.9

Values represent average concentration of indicated drug ± standard deviation (n = 3 rabbits for each drug dose)

SUPPLEMENTAL METHODS

Serum antibiotic concentration analyses. Rabbit serum concentrations of cefazolin and oxacillin after a 100 mg/kg i.m. dose were measured using a radial diffusion assay (1). Rabbit blood was collected intravenously 1 h and 2 h post drug administration, and serum was collected via centrifugation. *Bacillus subtilis* strain ATCC6633 was grown overnight in Brain Heart Infusion (BHI) medium and washed twice with PBS. Cells were resuspended in PBS, briefly sonicated, and adjusted to an optical density of OD_{600nm} 0.5 (~1 x 10^8 CFU/mL). Cells were diluted to a final concentration of 5 x 10^5 CFU/mL in MHA and poured into petri plates. A radial diffusion punch was used to make wells in the agar plates. Rabbit serum was diluted in a 2-fold dilution series in water, and 60 µL of each dilution was added to the agar wells. A standard plate was also prepared with cefazolin or oxacillin concentrations ranging from 0.0625 – 256 µg/mL in 60 µL water. The plates were incubated at 37°C for 3 h, after which the serum and drug standards were aspirated out of the wells and 10 mL of TSA was poured over the tops of the plates. Plates were incubated at 37°C overnight, after which the zones of clearance around the wells were measured. A standard curve was constructed and used to calculate rabbit serum concentrations of cefazolin and oxacillin.

Blood [HCO_3^-] analyses. The CG4+ i-STAT cartridge system (Abbott Point of Care Inc., New Jersey) was used as per manufacturer's instructions to measure [HCO_3^-] concentrations in whole rabbit blood collected from the lateral ear vein. Blood samples were taken from animals at baseline pre-infection, at 24 h post-induction of IE for untreated animals, or after 2 days of β-lactam treatment of animals with IE. Strains MRSA 11/11, MW2, COL, and BMC1001 were used for these studies.

REFERENCES

1. Bonev B, Hooper J, Parisot J. 2008. Principles of assessing bacterial susceptibility to antibiotics using the agar diffusion method. J Antimicrob Chemother 61:1295-1301.
